# Magnitude and associated factors of early Implanon discontinuation among rural women in public health facilities of central Ethiopia: a community-based cross-sectional study

**DOI:** 10.1186/s12905-022-01651-y

**Published:** 2022-03-14

**Authors:** Mekonnen Obsu, Berecha Hundessa, Sileshi Garoma, Haji Aman, Hailu Merga

**Affiliations:** 1 South West Shewa Zone Health Department, Oromia Region, Ethiopia; 2 USAID JSI, Addis Ababa, Ethiopia; 3 Department of Public Health, Adama Hospital Medical College, Adama, Ethiopia; 4 Department of Epidemiology, Institute of Health, Jimma University, Jimma, Ethiopia

**Keywords:** Early discontinuation, Implanon, Rural women, Central Ethiopia

## Abstract

**Background:**

Advocating for and promoting contraception use is critical in reducing mother and child morbidity and mortality. Early Implanon discontinuation may result in unwanted pregnancies and induced abortions. Although there has been research in Ethiopia on long-acting reversible contraceptives, there has been none on early Implanon removal among rural women who have used and removed it. Hence, this study aimed to investigate the magnitude of Implanon discontinuation and related characteristics among women who had the Implanon removed in central Ethiopian public health facilities.

**Methods:**

A community-based cross-sectional study was conducted among all women of the reproductive age group who had removed Implanon after using it. A simple random sampling technique was used to select 373 women after proportional allocation to each health facility. Data were collected using a pretested semi-structured interviewer-administered questionnaire adapted from previous studies. Data were entered using EpiInfo and exported to SPSS version 21 for analysis. A binary logistic regression model was used to determine the association between the outcome variable and independent variables. A p-value less than 0.25 was used as a cutoff point to select candidate variables for the final model. Then, a p value less than 0.05, AOR, and a 95% confidence level were used to declare statistical significance.

**Result:**

A total of 360 participants responded to the questionnaires, making a response rate of 96.5%. In this study, the early discontinuation rate was 42% (95% CI 36.9–47.7). No formal education (AOR = 0.53 [95% CI 0.3–0.94], having medium monthly income (AOR = 3.02 [95% CI 1.38–6.6]), inadequate pre-insertion counseling (AOR = 0.55 [95% CI 0.31–0.98]), lack of appointment for follow up (AOR = 0.16 [95% CI 0.05–0.54]), didn`t satisfy with service provided (AOR = 0.067 [95% CI 0.015–0.29] and developed side effect (AOR) = 4.45 [95% CI 2.37–8.36] were significantly associated with Implanon discontinuation.

**Conclusion:**

The discontinuation rate of Implanon among those who removed it after using it in this study was high. Lack of formal education, having a medium-income, inadequate pre-insertion counseling, lack of appointments for the follow-up, poor satisfaction, and problems with side effects were the factors associated with early discontinuation rate. Hence, quality family planning service provision is essential to reduce the discontinuation rate.

**Supplementary Information:**

The online version contains supplementary material available at 10.1186/s12905-022-01651-y.

## Background

Unplanned pregnancies constitute a global problem associated with substantial costs to health and social services as well as significant emotional distress to women and their families. Hence, the promotion of contraception utilization is of paramount importance in reducing poverty and hunger as well as maternal and child morbidity and mortality. Globally, about 86 million pregnancies were unintended, of which 33 million resulted in unplanned births [1, 2]**.**

Implanon, small plastic rods each about the size of a matchstick, is provided by the specifically trained provider by performing a minor surgical procedure and is very effective contraception [3–5]. Early Implanon discontinuation is defined as discontinuation at less than two and half years after the insertion of Implanon [6, 16, 21]. Worldwide, the magnitude of women's satisfaction with modern family planning methods increased from 75% in 2000 to 78% in 2017 [7]. Implants are the most widely used method by married women in sub-Saharan African countries [8]. Providers need to discuss the likelihood, unpredictability, and side effects, exploring their acceptability given the client's circumstances and socio-cultural context [9]. Contraceptive use among East African women, Ethiopia, Kenya, and Rwanda in particular, has increased over the past two decades [10].

In Ethiopia, contraceptive use has shown a tremendous increase in the last decades, following the launching of the health extension program. Although nearly one-third of women still prefer to delay their next birth for at least 2 years, only a small number of women are currently using long-acting reversible contraceptive methods [4]. The Ethiopian Demographic Health Survey showed that the use of implants was 8% in 2016 [5]. In Ethiopia, the magnitude of early discontinuation varies: 25.5% from South west Ethiopia [11], 19% from Northern Ethiopia [12], 23.4% from Southern Ethiopia [13], and 50% from Arsi, Oromia region [14].

Although there are studies on long-acting reversible contraceptives in Ethiopia, to the best of our knowledge, there is no study on early Implanon removal among mothers who removed it. Most of the studies focused on the urban population and overall contraceptive discontinuation rather than focusing on specific contraceptive discontinuation and rural mothers, as more than 80% of the Ethiopian population lives in rural areas. Therefore, this study assessed the early Implanon discontinuation rate and associated factors among rural women who removed the Implanon after using it, which will help to increase the continuation rate of Implanon users and improve future national family planning programs.

## Methods and materials

### Study design and setting

A community-based cross-sectional study was conducted using quantitative data collection methods from June 1 to July 30, 2019. The study was conducted in Woliso rural Woreda which is found in South West Shoa, Oromia regional state of Ethiopia. Based on the 2007 National Census conducted by the Central Statistical Agency of Ethiopia (CSA) projection, the total population of Woreda was 192,757. Among these, 13,969 were in the reproductive age group. There were 8 health centers and 35 health posts in the woreda.

### Population and sampling

**Fig. 1:**
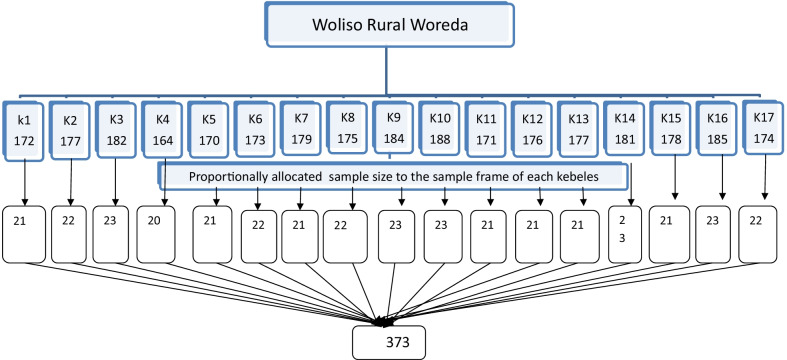
Diagrammatic presentation of sampling procure (k = kebele)

All women in the reproductive age range (15–49) who used Implanon and removed it within the past 3 years were included in our study. A single population proportion formula was used to calculate the sample size for this study. Accordingly, the proportion of early discontinuation of Implanon was 46.5% which was taken from the previous study [15], 95% level of confidence, and 5% margin of error. Since the source population (2989) in the study area was less than 10,000, we used the population correction factor and with 10% nonresponse rate, the final sample size became 339. Of the 35 Kebeles found in the district, 17 Kebeles were randomly selected and included in the study. House-to-house interviews were conducted in each kebele among the respondents using simple random selection after the lists of those women were taken from each health facility registration book. Repeat household visits were made for women who were not at home during the visit to reduce the non-response rate, and those who refused to respond were recorded as non-respondents (Fig. 1).

### Data collection tool and procedure

Data were collected using a semi-structured face-to-face interviewer administered questionnaire developed after reviewing different literature [16] (Additional file 1). It was prepared in English and translated into regional working language (Afaan Oromoo) and then retranslated back to English by different language experts to check for consistency. Ten health extension workers and two midwifery degree holders participated in the data collection and supervision respectively. To ensure the quality of data, they were trained for 2 days, the tool was also pretested on 5% of the sample on one kebele of the neighboring district, and appropriate modifications were made. The collected data were reviewed and checked for completeness every day before data entry.

### Study variables

#### Dependent variable

Early Implanon discontinuation.

#### Independent variables

- Socio-demographic character: Marital status, religion, occupation, and education.
- Obstetric factors: number of children, parity, and abortion.
- Social factors: husband objection, husband involvement, and husband go abroad and Neighbors influence
- Method related factors: side effects, past contraceptive utilization, desire for pregnancy, follow-up, counseling and weight gain

### Operational definition

Early Implanon discontinuation is the removal of Implanon by health professionals before 2.5 years of utilization [6, 16, 21].

### Data analysis

STROBE checklist was used to analyze and report data [17]. Data were checked, coded, and entered into EpiInfo and then, exported to SPSS version 21 for analysis. Both descriptive and analytical statistical procedures were utilized. Descriptive statistics like percentage, mean, median, and standard deviation were used for the presentation of socio-demographic data and the prevalence of early discontinuation of Implanon. Binary logistic regressions were used to identify factors associated with early discontinuation of Implanon. Variables with a p-value < 0.25 on the bi-variable analysis were entered into multiple logistic regression models to identify their independent effects on the outcome variable. Model fitness was checked using the Hosmer and Lemeshow goodness of fit test. All the assumptions like the normality of continuous variables and multicollinearity of independent variables were checked to be satisfied using Variance Inflation Factors (VIF). Multiple logistic regression models were used to control the possible effect of confounders. Variables that have an independent association with early discontinuation of Implanon were identified based on OR with its 95% CI and p-value less than 0.05 were used to declare the level of significance.

## Results

### Socio-demographic characteristics of respondents

A total of three hundred sixty (360) participants have responded to the questionnaires, making a response rate of 96.5%. The age of study participants was between 16 and 46 years with a mean (+ SD) age of 29.1 ± 6.15 years. Almost all of the participants 357 (99.2%) were married and near to two-thirds 234 (65%) of them were Orthodox Christians. By their occupation, more than three fourth 304 (84.4%) of them were housewives. Two hundred fifty-six (71.1%) of those respondents had children between one and three, with a mean (+ SD) of 2.8 ± 1.6. The majority of the study participants 167 (46.4%) and more than half 210 (58.3%) of their husbands attended primary education (Table 1).

**Table 1:** Sociodemographic status of women who ever used Implanon in the last 3 years in Woliso District, Oromia, central Ethiopia, 2019 (n = 360)

Variables	Characters	Frequency (%)
Women’s Age at the time of Implanon insertion	< 20	31 (8.6)
	20–25	84 (23.3)
	26–30	116 (32.2)
	31–35	60 (16.7)
	> 35	69 (19.2)
Marital status	Married	357 (99.2)
	Single	3 (0.8)
Religion	Orthodox	234 (65.0)
	Protestant	104 (28.9)
	Muslim	22 (6.1)
Women’s Educational status	Unable to read and write	125 (34.7)
	Primary	167 (46.4)
	Secondary	49 (13.6)
	Diploma and above	19 (5.3)
Husband’s educational status	Unable to read and write	59 (16.4)
	Primary	210 (58.3)
	Secondary	55 (15.3)
	Diploma and above	36 (10.0)
History of abortion	No	328 (91.1)
	Yes	32 (8.9)
Women’s occupation	Housewife	304 (84.4)
	Student	2 (0.6)
	Teaching	7 (1.9)
	Private own business	30 (8.3)
	Employee	16 (4.4)
Living children	0	14 (3.9)
	1–3	256 (71.1)
	4–6	80 (22.2)
	> 6	10 (2.8)

### Past contraceptive history and counseling status during Implanon insertion

Three hundred fifty-four (98.3%) of the participants had ever heard of any contraceptive before inserting Implanon, and among these 297 (82.5%) of the participants reported that they knew the benefit of Implanon, 198 (55%) its side effects, and 180 (50%) also knew its duration of action. The main sources of information for the participants were mainly Health workers 280 (77.8%) followed by their neighbors 45 (12.5%). Less than one-quarter of the participants, 66 (18.3%), didn't use any type of modern contraceptive before inserting the Implanon and the rest, 294 (81.7%) had used a modern method of contraceptives. Injectables 262 (72.8%) and pills 27 (7.5%) were the major modern contraceptive methods employed. Almost all of the participants (99.2%) got counseling services with 262 (72.8%) individually, 162 (45%) mass, and 45 (12.5%) with husband counseling. Among those who got counseling service during insertion, 311(86.4%) were counseled about the benefits, 282 (78.3%) about when to insert and remove, 232 (64.4%) about its effectiveness, and 218 (60.6%) were also about its side effects and 199 (55.3%) of participants reported they got counseling about the duration of action of Implanon. Near two-thirds, 232 (64.4%) and more than one-third 128 (35.6%) of the participants received the Implanon insertion from health centers and health posts respectively (Table 2).

**Table 2:** Past contraceptive history, counseling status of women who discontinued Implanon within last 3 years Woliso district, Oromia, central Ethiopia 2019

Variables	Characters	Frequency (%)
Information of ever heard about any contraception before using Implanon (n = 360)	No	6 (1.7)
	Yes	354 (98.3)
Type of information they know about Implanon before inserting (some clients had more than one response)	Benefit	297 (82.5)
	Effectiveness	256 (71.1)
	Side effects	198 (55.0)
	Duration of action	181 (50.30)
Ever used any contraceptive before using Implanon (n = 360)	No	66 (18.3)
	Yes	294 (81.7)
Type of contraceptive they used before inserting Implanon (last method n = 294)	Pills	27 (7.5)
	Injectables	262 (72.8)
	Others	5 (1.4)
Counseling service during inserting Implanon	No	3 (0.8)
	Yes	357 (99.2)
Type of counseling (had more than one response)	Individual counseling	262 (72.8)
	Mass counseling	162 (45.0)
	With husband counseling	45 (12.5)
Type of information obtained during counseling (some clients had more than one response)	Duration of action	199 (55.3)
	Advantage	311 (86.4)
	Side effects	218 (60.6)
	Effectiveness	232 (64.4)
	When to insert and remove	282 (78.3)
	Others	1 (0.3)
Source of information	Health workers	280 (77.8)
	Neighbors	45 (12.5)
	Friends	10 (2.8)
	Mass media	3 (0.8)
	School	16 (4.4)

They said that they preferred using Implanon mainly due to the long time 119(33.1%), convenient to use 86 (23.9%) and for birth spacing 82 (22.8%). Almost near to half of the women 155 (43.1%) have developed side effects after inserting Implanon. Menstrual disorder 64 (17.8%), headache 53 (14.7%), dizziness 28, (7.8%), and weight loss 7, (1.9%) were among the women who reported perceived side effects, along with others such as back pain and generalized body rash. One-third 122 (33.9%) of the women reported irregular menses during the use of Implanon. Similarly, nearly one-third of the women (31.7%) had an overall decrease in menstrual blood loss. Also, 336 (93.3%) of the participants were appointed at a specific time for follow-up after Implanon insertion, 330 (91.7%) were satisfied with the service given during the insertion.

### Early discontinuation of Implanon

Of the 360 women who removed Implanon after using it in the last three years, the early Implanon discontinuation rate was 42% (95% CI 36.9–47.7) and they had used it for 3 to 30 months, with a mean of 26.78 months. The majority 39.2% of the discontinuers had used Implanon for less than two years, followed by less than 12 months (22.2%) and before 6 months (7.2%). Even though husband opposition and inconvenience of use were less frequent reasons, mainly they discontinued due to its side effect 100 (48.3%), desire for pregnancy 98 (47.3%), and method shift 9 (4.3%). The common side effects for discontinuation of Implanon were menstrual disruption 64 (17.8%) and Headache 53 (14.7%). Among the women who had discontinued by shifting to other methods, 102 (75%) and 17 (12.5%) were shifted into Injectable and IUCD respectively.

### Factors associated with early Implanon discontinuation

All the socio-demographic and counseling relating factors were assessed for the presence of association with an early discontinuation rate of Implanon in bi-variable analysis. Hence, age, women and husbands’ educational level, length of counseling before insertion, income, satisfaction by the service, appointed for follow up, side effect, having children during insertion,

having living children were the candidate variables for the final model using p-value less than 0.25 as the cut off points. Then, multivariable logistic regression analysis was done and found that development of side effects after the insertion of Implanon, appointment for follow up, women`s educational level, length of counseling before insertion, income, and satisfaction with the service given during Implanon insertion were statistically significant.

The study demonstrated that the odds of early Implanon discontinuation among women who developed the side effects were 4.45 times than those who did not develop the side effects (AOR) = 4.45 [95% CI 2.37–8.36]. Women who were appointed for follow-up were 84% less likely to discontinue Implanon as compared with those who had not followed up after insertion (AOR = 0.16 [95% CI 0.05–0.54]. The odds of Implanon discontinuation among women who attended the primary level of education were 47% less likely than those who were illiterate (AOR = 0.53 [95% CI 0.3–0.94]. Women earning 500–1000 Eth birr per month were three times more likely to discontinue the methods than women earning less than 500 Eth birr per month (AOR = 3.02 [95% CI 1.38–6.6]). Women who got more time on counseling discontinued Implanon early 45% less likely compared to those who got less time of counseling before insertion (AOR = 0.55 [95% CI 0.31–0.98]. Women who spent more time on counseling before insertion were 45% less likely to discontinue Implanon earlier than those who spent less time on counseling before insertion (AOR = 0.55 [95% CI 0.31–0.98]. Women who were satisfied with the service given during the insertion of Implanon were 93% less likely to discontinue Implanon early as compared with those who were not satisfied during the insertion (AOR = 0.067 [95% CI 0.015–0.29] (Table 3).

**Table 3:** Factors associated with Early Implanon discontinuation among women who discontinued Implanon within last 3 years Woliso district, Oromia, central Ethiopia 2019

Variables	Early Implanon discontinuation		COR (95% C.I)	AOR (95% C.I)
	No (%)	Yes (%)		
*Women educational level*				
Illiterate	70 (56%)	55 (44%)	1	1
Primary	114 (68.3%)	53 (37.7%)	0.59 (0.366.0.957)	0.534 (0.302, 0.944)*
Secondary	19 (38.8%)	30 (61.2%)	2.01 (1.024, 3.945)	0.885 (0.346, 2.26)
Diploma and above	6 (31.6%)	13 (68.4%)	2.758 (0.985, 7.723)	1.04 (0.237, 4.556)
*Income*				
< 500 Eth Birr	110 (71.9%)	43 (28.1%)	1	
500–1000 Eth Birr	39 (45.3%)	47 (54.7%)	3.083 (1.775, 5.353)	3.017 (1.379, 6.602)**
> 1000 Eth Birr	60 (49.6%)	61 (50.4%)	2.6 (1.575, 4.293)	1.373 (0.64, 2.947)
*Length of counseling*				
< 30 min	65 (48.5%)	69 (61.5%)	1	1
30–60 min	68 (59.1%)	47 (40.9%)	0.65 (0.394, 1.077)	0.89 (0.516, 1.537)
> 60 min	76 (68.5%)	35(31.5%)	0.434 (0.257, 0.733)	0.548 (0.308, 0.975)*
*Side effect after*				
No	158 (77.1%)	47 (22.9%)	1	1
Yes	51 (32.9%)	104 (67.1%)	6.855 (4.296, 10.94)	4.454 (2.37, 8.36)**
*Appointment*				
No	7 (29.2%)	17 (70.8%)	1	1
Yes	202 (60.1%)	134 (39.9%)	0.273 (0.11, 0.676)	0.163 (0.049, 0.538)**
*Satisfaction*				
No	3 (10%)	27 (90%)	1	1
Yes	206 (62.4%)	124 (37.6%)	0.067 (0.02, 0.225)	0.067 (0.015, 0.29)**

*Significantly associated at p-value < 0.05

**Highly significantly associated at p-value < 0.001

## Discussion

The proportion of early Implanon discontinuation among women who removed Implanon in public health facilities was 42% with a mean duration of 26.78 months. The result is higher than studies in Nigeria (26.1%) [18] but lower than the studies conducted in Debre Tabor (65%) [16] and Arsi zone, Ethiopia (80%) [14]. This discrepancy might be due to sample size, socio-cultural differences, and governmental implementations done to minimize early removal of Implanon.

This study showed that the discontinuation rate was 7.2% within 6 months, 22.2% within 1 year, 39.2% within 2 years, and 45% up to 3 years. This result is higher than the study done in Senegal in which the removal rate was 3.3% at 6 months, 6.3% at 12 months, 10% at 18 months, and 15% at 24 months [19]. But our study result was lower than the study done in South Africa, 27.2% of the participants removed the Implanon in the first six months of use, 67.3% removed in the first year of use while 94.4% had removed it after the second year [20] and also lower than in Debre Markos which was documented as 22.6% discontinued within 6 months, 51.4% within 12 months and 82.2% within 24 months [15]. The reason for this discrepancy might be the difference in counseling service given during the inception of the mothers into the method and continuous follow-up on those mothers. For instance, in this study, 60% and 64% of the participants were counseled on the side effect and effectiveness of the contraceptive methods respectively, whereas in the study done in Debre Markos 54% and 53% of the participants were counseled each. Other factors such as study denominators, sample size, and the socio-cultural difference might be a reason.

The main reasons cited by the women for early discontinuation of Implanon were side effects 100 (48.3%) and desire for pregnancy 98 (47.3%). This is consistent with other studies conducted in Port Harcourt and Ethiopia [21, 22]. The most common side effects for discontinuation of Implanon were 64 (17.8%) menstrual disruption and Headache 53 (14.7%). This is lower than two studies from Nigeria and Ethiopia [18, 21–23]. It might be due to adequate counseling before insertion that having prior information on the expected side effects of the method and tolerance of the side effects. The main sources of information for the participants were Health workers, which is in agreement with studies from South Africa and Ethiopia [24, 25].

This study revealed that women who had relatively medium monthly income 500–1000 Ethiopian Birr) were 3 times more likely to discontinue the methods compared to those who were earning less 500 Ethiopian Birr (AOR = 3.02[95% CI 1.38–6.6]. This is contrary to the study done in Ethiopia (Dale District) that Women who had relatively high monthly income were less likely to discontinue the methods compared to those who earn less [26]. This might be due to those who had better income need more children because they thought that they could give the necessary care and grow children easily than their counterparts. On the another hands, they respond easily to minor side effects and discontinue it since they might have access to transportation and remove with payment at private facilities.

The odds of Implanon discontinuation among women who attended the primary level of education were 47% less likely than the odds of discontinuation in those who were illiterate (AOR = 0.53 [95% CI 0.3–0.94]. This might be due to the benefit of education to understand and outweigh the advantages and disadvantages of the methods they are using. This finding is also in line with the studies done in Ofla and Diguna Fango that showed Implanon discontinuation was more common among women not formally educated [21, 27].

Women who had enough pre-insertion counseling on Implanon were 45% less likely to discontinue as compared to those who hadn`t enough counseling or were counseled for less than 30 min of duration (AOR = 0.55 [95% CI 0.31–0.98]. Lack of proper counseling and information regarding the side effects, method change was more likely to result in a negative attitude towards methods whenever they experienced the side effects. The possible explanation could be if women have very clear information about the provided service, they will give up with minor side effects and may have a higher chance of continuing the method. This finding is also in line with studies done in Ofla, northern Ethiopia [27].

Women who were appointed for follow-up were 84% less likely to discontinue the Implanon as compared to those who had no appointment for follow-up. This might be during follow-up visit, if any compliant comes from the client, they will get an appropriate solution from the health workers, and also there might be post insertion counseling on the expected side effects specific to Implanon. This finding is also similar to studies done in Southern Ethiopia [28].

Similar to other previously done studies, this study also showed that mothers who experienced Implanon side effects were four-folds more likely to remove the methods early as compared to those who didn't experience a side effect [AOR = 95% CI 4.45 (2.37, 8.36)]. This result is in line with the study done in Southern and Northern Ethiopia [28, 29]. This might be due to the fact that side effects directly affect mothers` tolerance and use which precipitates them to change the method or withdraw from Implanon use. On the other hand, the family planning provider's gap in skill and knowledge concerning the management of side effects could increase the discontinuation habit of those mothers in need of removal.

Women who were satisfied with the service given during the insertion of Implanon were 93% less likely to discontinue Implanon as compared to those who were not satisfied with the service given during the insertion of Implanon (AOR = 0.067 [95% CI 0.015–0.29]. This is because women who were interested in the method choice, privacy, explanation of the service provider, and other service provisions during the insertion of the Implanon may be interested in continuing using the method. It is similar to the study from Northern Ethiopia and Southern Ethiopia [27, 28].

## Conclusion

In this study the prevalence of early discontinuation of Implanon was high. Women’s educational status, women’s income, women who developed side effects during using Implanon, women who didn’t get appointments for follow up, and women who were not satisfied with the given service during the Implanon insertion were the predictors of early Implanon discontinuation. As a result, at different hierarchical level of the health system, significant efforts should be undertaken to address women's perception and knowledge through mass media and health education programs in order to raise the percentage of implanon retention. Health care practitioners should also offer adequate pre-insertion counseling to clients based on the handbook, with an emphasis on adverse effects. Furthermore, implanon users should be closely monitored and followed up on to enhance the rate of implanon continuance.

## Supplementary Information

**Additional file 1**: Data collection tool.

## Data Availability

All data generated or analyzed during this study are included in this article.

## Abbreviations

CSA: Central Statistical Agency
CI: Confidence Interval
EDHS: Ethiopian Demographic and Health Survey
FMOH: Federal Ministry of Health
HEW: Health Extension Worker
LAFP: Long-Acting Family planning
OR: Odds Ratio
SPSS: Statistical Package for Social Sciences
WHO: World Health Organization
