# Supplementary material for: Magnitude and associated factors of early Implanon discontinuation among rural women in public health facilities of central Ethiopia: a community-based cross-sectional study
Source: BMC Womens Health. 2022 Mar 14;22:72. doi: 10.1186/s12905-022-01651-y (PMC8922880; doi:10.1186/s12905-022-01651-y)
Supplement: Supplementary file 1 — Additional file 1: Data collection tool. [file 12905_2022_1651_MOESM1_ESM.docx]

**Data collection tool**

**Instruction**: - Circle the responses for questions with alternatives and for open ended questions write on the space provided.

**Part I: Socioeconomic and Demographic characteristics**

| QN | **Questions and filter** | **Coding categories** | **Skip** |
| --- | --- | --- | --- |
| 101 | What was your age at time of insertion? | Age ……………in years |  |
| 102 | What was your marital status at time of insertion? | Single……………………………………….1  Married …………………………………….2  Widowed …………………………………..3  Divorced /separated ………………………4 |  |
| 103 | Have you ever attended a School? | Yes …………………………………………1  No ………………………………………….2 | If No skip to  105 |
| 104 | What is the highest grade you completed? | Grade............... |  |
| 105 | Have your husband ever attended a School? | Yes …………………………………………1  No ………………………………………….2 | If No skip to  107 |
| 106 | What is the highest grade your husband completed? | Grade……….. |  |
| 107 | What is your religion? | Orthodox…………………………………1  Muslim …………………………………..2  Protestant…………………………………3  Others (**specify**) …………………………… |  |
| 108 | What is your main occupation? | …………………… |  |
| 109 | How much is your Monthly income(**Birr**) | …………………… |  |

| QN | **Questions and filter** | **Coding categories** | **Skip** |
| --- | --- | --- | --- |
| 201 | Did you have children during insertion of implanon? | Yes ………………………1  No ………………………2 | If No skip  to **204** |
| 202 | If **yes** how many children did you have? | …………………. |  |
| 203 | How many more children do you want to have? | …………………… |  |
| 204 | Do you intend to have children? | Yes ………………………1  No ………………………2 | If No skip  to **206** |
| 205 | If **yes** how many do you want to have? | ……………………….. |  |
| 206 | Did you have any history of abortion? | Yes ………………………1  No ………………………2 | If No skip to  **301** |
| 207 | If **yes** how many times? | ……………………. |  |

**Part –II Gynecological History**

**Part III Past knowledge and utilization of contraceptive methods**

| QN | **Questions and filter** | **Coding categories** | **Skip** |
| --- | --- | --- | --- |
| 301 | Have you ever heard information about contraceptive methods? | Yes ……………………1  No ……………………2 | **If No skip**  **to** 3**04** |
| 302 | From where did you obtain the information? | ………………………………. |  |
| 303 | What type of information did you know?  (**Multiple answers possible**) | Effectiveness ……………………1  Side effects ………………………2  Duration of action ………………3  Benefit ………………………4  Others (specify) ……………… |  |
| 304 | Have you ever used contraception before using Implanon? | Yes ………………………………1  No ………………………………2 | If No skip to  **306** |
| 305 | Which method you did use? (**last method**) | Pills ………………………………1  IUCD ……………………………2  Injectables ...……………………3  Others (**specify**)………………… |  |
| 306 | What was the main reason for not using any  Contraceptive method before? | …………………………………… |  |

**Part IV Role of partner and counseling services**

| QN | **Questions and filter** | **Coding categories** | **Skip** |
| --- | --- | --- | --- |
| 401 | Did you get counseling service before  Inserting the implanon? | Yes ……………………1  No ……………………2 | If No skip to  **406** |
| 402 | What type of counseling did you obtain? | Individual counseling…1  Mass counseling ………2  With husband counseling …………….3  Other (**specify**) ………… |  |
| 403 | For how long did you get the counseling  service |  |  |
| 404 | After how long the counseling service of did you inserted implanon? | ………………. |  |
| 405 | What type of information did you obtain during the counseling?  (**Multiple answers possible**) | Advantage ………………1  Side effects ………………2  Duration of action ………3  Effectiveness ……………4  When to insert and remove ……..…….5  Other (**specify**) …………… |  |
| 406 | Did you first discuss with your partner Exactly to use this method of FP? | Yes……………………………………..1  No ……………………………………..2 | If “yes” skip  to 4**08** |
| 407 | Why not you discuss with your husband? |  |  |
| 408 | Did he accept at that time? | Yes……………………………………1  No ……………………………….……2 |  |
| 409 | Who was decided inserting the Implanon? | …………………… |  |
| 410 | When did you insert the Implanon? | ……/……./2019 E.C |  |
| 411 | Where did you insert Implanon | ……………………………….. |  |
| 412 | Why did you choose to use Implanon? | ………………………………… |  |
| 413 | Did you feel any side effect after inserting implanon? | Yes …………………………………1  No …………………………………2 | If No skip to  **415** |
| 414 | If yes, what type of side effect(s)? | …………………………… |  |
| 415 | How it comes your menstrual cycle after you have started using implanon? | Regularly …………………………1  Irregular ……………………………2  It already stops…………..…………3 | If it is already  stops skip to  **417** |
| 416 | Was there any change in the amount of menstrual loss after you have started using implanon? | No change ……………………………..1  Increased …………………………2  Decreased ………………………3  Others (**specify**)……………………… |  |
| 417 | After the insertion of implanon, did they appoint you at a specific time? | Yes …………………………………1  No …………………………………2 |  |
| 418 | Did you satisfy by the service given to you by the FP service providers during the insertion? | Yes ……………………………………1  No …………………………………2 |  |

**Part-V Reasons for removal of Implanon use**

| QN | **Questions and filter** | **Coding categories** | **Skip** |
| --- | --- | --- | --- |
| 501 | When did you remove the implanon? | ……../……./…….E.C |  |
| 502 | For how long did you use implanon? | …………………….. |  |
| 503 | Did you discontinue early? | Yes………………..1  No……………….2 |  |
| 504 | What was the reason you had remove using of implanon? | ……………………… |  |
| 505 | If the reason was shifting to other method, which method is using currently? | Pills …………………………1  IUCD ………………………2  Injectables ...…………………3  Others (**specify**)……………… |  |
